# Kidney failure, CKD progression and mortality after nephrectomy

**DOI:** 10.1007/s11255-022-03114-7

**Published:** 2022-01-27

**Authors:** Robert J. Ellis, Anne Cameron, Glenda C. Gobe, Vishal Diwan, Helen G. Healy, Jeremy Lee, Ken-Soon Tan, Sree Venuthurupalli, Jianzhen Zhang, Wendy E. Hoy, the NHMRC CKD.CRE, the CKD.QLD Collaborative

**Affiliations:** 1 Princess Alexandra Hospital, Brisbane, QLD Australia; 2 Faculty of Medicine, University of Queensland, Brisbane, QLD Australia; 3 Kidney Disease Research Collaborative, Translational Research Institute, 37 Kent Street, Woolloongabba, Brisbane, QLD 4102 Australia; 4 NHMRC CKD.CRE and the CKD.QLD Collaborative, University of Queensland, Brisbane, QLD Australia; 5 Kidney Health Service, Royal Brisbane and Women’s Hospital, Brisbane, QLD Australia; 6 Conjoint Internal Medicine Laboratory, Pathology Queensland, Brisbane, QLD Australia; 7 Department of Nephrology, Logan Hospital, Logan, QLD Australia; 8 Renal Service, Ipswich Hospital, Brisbane, QLD Australia

**Keywords:** Chronic kidney disease, Nephrectomy, Kidney failure

## Abstract

**Purpose:**

This study tested the hypothesis that progression of chronic kidney disease (CKD) is less aggressive in patients whose primary cause of CKD was nephrectomy, compared with non-surgical causes.

**Methods:**

A sample of 5983 patients from five specialist nephrology practices was ascertained from the Queensland CKD Registry. Rates of kidney failure/death were compared on primary aetiology of CKD using multivariable Cox proportional hazards models. CKD progression was compared using multivariable linear and logistic regression analyses.

**Results:**

Of 235 patients with an acquired single kidney as their primary cause of CKD, 24 (10%) and 38 (17%) developed kidney failure or died at median [IQR] follow-up times of 12.9 [2.5–31.0] and 33.6 [18.0–57.9] months after recruitment. Among patients with an eGFR < 45 mL/min per 1.73m^2^ at recruitment, patients with diabetic nephropathy and PCKD had the highest rates (per 1000 person-years) of kidney failure (107.8, 95% CI 71.0–163.8; 75.5, 95% CI 65.6–87.1); whereas, patients with glomerulonephritis and an acquired single kidney had lower rates (52.9, 95% CI 38.8–72.1; 34.6, 95% CI 20.5–58.4, respectively). Among patients with an eGFR ≥ 45 mL/min per 1.73m^2^, those with diabetic nephropathy had the highest rates of kidney failure (16.6, 95% CI 92.5–117.3); whereas, those with glomerulonephritis, PCKD and acquired single kidney had a lower risk (11.3, 95% CI 7.1–17.9; 11.7, 95% CI 3.8–36.2; 10.7, 95% CI 4.0–28.4, respectively).

**Conclusion:**

Patients who developed CKD after nephrectomy had similar rates of adverse events to most other causes of CKD, except for diabetic nephropathy which was consistently associated with worse outcomes. While CKD after nephrectomy is not the most aggressive cause of kidney disease, it is by no means benign, and is associated with a tangible risk of kidney failure and death, which is comparable to other major causes of CKD.

**Supplementary Information:**

The online version contains supplementary material available at 10.1007/s11255-022-03114-7.

## Introduction

Kidney reduction surgery, removal of all (radical/total nephrectomy) or part (partial nephrectomy) of the kidney, is the most common management approach for suspicious kidney masses, and curative for most localised kidney cancers [1]. While providing good oncological control, the surgical reduction of functional kidney parenchyma can contribute to reduced kidney function, characterised by declining estimated glomerular filtration rate (eGFR). Many patients with previously normal kidney function pre-nephrectomy will be classed as having chronic kidney disease (CKD) postoperatively, based on eGFR criteria. In the only randomised controlled trial comparing radical with partial nephrectomy, 85.7% of patients managed with radical nephrectomy and 64.7% of patients managed with partial nephrectomy had an eGFR < 60 mL/min per 1.73m^2^ (stage ≥ 3a CKD) at a median follow-up time of 6.7 years postoperatively [2]. While tumours are the most frequent indication for nephrectomy, other common indications include trauma, infection, obstruction, and donation.

Several authors have reported that, compared with ‘medical’ causes of nephron loss, surgical nephron reduction is associated with lower rates of CKD progression, kidney failure, and death [3, 4]. One interpretation is that medical kidney disease is associated with continuous exposure to the underlying pathophysiology causing nephron loss, whereas the surgical insult is time-limited and, presumably, not associated with chronic pathophysiological processes [5]. This interpretation is not grounded in evidence. The cohort of medical causes of CKD is extremely broad, with considerable heterogeneity in the cause of kidney disease, rates of CKD progression, and severity of kidney impairment [6]. Many patients with CKD will not experience obvious declines in kidney function over time or progress to kidney failure [7–9]. Notwithstanding, all patients with stages 3b, 4 and 5 CKD have higher risks of mortality compared with patients who have stages 1, 2 and 3a [10], and this risk is inversely proportional to eGFR [11].

This study aimed to test the hypothesis that rates of kidney failure, mortality, and CKD progression are lower in patients whose CKD is due to surgical nephron reduction (acquired single kidney) compared with other medical causes, within a population of pre-dialysis patients known to specialty nephrology services in Queensland, Australia.

## Patients and methods

### Study population

Data were extracted for 5983 patients enrolled in the Queensland CKD Registry (CKD.QLD) from five clinical sites across Queensland, Australia: Royal Brisbane and Women’s Hospital (*n* = 1643), Logan Hospital (*n* = 1534), Mackay Base Hospital (*n* = 680), Toowoomba Hospital (*n* = 1470), and Townsville University Hospital (*n* = 656) [12]. Patients were recruited between June 2011 and June 2018; data were extracted from the registry in January 2020. Inclusion criteria for the registry are a diagnosis of CKD and care under a specialist nephrology practice in Queensland.

Patients were excluded if they had an unclear, unknown, or unspecified primary kidney disease (*n* = 625), dates were ambiguous or missing (*n* = 106), baseline eGFR data were missing (*n* = 27), or if they had kidney failure (eGFR < 15 mL/min per 1.73m^2^) at the time of recruitment (*n* = 345). There were 4,880 patients in the final sample. Human research ethics approval was obtained from the Royal Brisbane and Women’s Hospital (HREC/15/QRBW/294) and the University of Queensland (2011000029) and a Public Health Act Approval (QCOS/029817/RD006802) for Queensland Health data linkages. All participants provided written informed consent before being included in the CKD.QLD registry.

The registry is informed by an association with the Statistical Analysis and Linkage Unit (SALU), Queensland Health, who link the CKD.QLD registry patient cohort to the following collections under a Public Health Act approval: Queensland Hospital Admitted Patient Data Collection (QHAPDC), Kidney replacement therapy (KRT), Queensland Activity Based Funding (ABF) Model Output Data, Death Registrations from the (Qld) Registry of Births, Deaths and Marriages and Cause of Death Unit Record File from the Australian Coordinating Registry, informing on institution of KRT and deaths.

### Exposures

Age at recruitment was calculated from the date of birth and date of recruitment. Date of recruitment was defined as the date informed consent was given for inclusion, capturing both incident and prevalent cases. Diabetes mellitus and hypertension were classified as yes, no, or missing. Smoker status was classified as never, former or current smoker, or missing. The primary kidney disease was grouped into five categories (diabetic nephropathy, glomerulonephritis, polycystic kidney diseases [PCKD], acquired single kidney, or other) based on coded diagnosis and free-text fields in the database, where the person’s treating nephrologist assigned the primary cause(s) of CKD based on clinical assessment. The acquired single kidney group included those where nephrectomy was listed as the primary cause of CKD. An ancillary chart review of this group was undertaken to determine the date and indication for nephrectomy. Baseline eGFR was determined from serum creatinine taken at the time of recruitment; eGFR at 12 and 24 months after recruitment was recorded if available in the patient’s electronic medical records. The CKD-EPI equation was used to calculate eGFR. Data collection has been described previously [12].

### Outcomes

The primary outcome of interest was a composite of time to the development of kidney failure (defined as eGFR persistently < 15 mL/min per 1.73m^2^) or commencement of KRT. Secondary outcomes included time to all-cause mortality, change in eGFR, and CKD progression. The CKD.QLD registry provided event data for kidney failure, KRT, and death, after performing data linkages of state electronic data sets. Change in eGFR was calculated over at least a 12-month period, and defined as the difference between baseline eGFR at recruitment, and eGFR at either 12 or 24 months, depending on the most recent value available. Progressive CKD was defined as an annual decrease in eGFR of at least 5 mL/min per 1.73m^2^ without subsequent recovery, the development of kidney failure, or the requirement for KRT within two years of recruitment.

### Statistical analysis

Baseline characteristics were presented descriptively. The association between primary kidney disease and time to kidney failure/KRT (primary outcome) and all-cause mortality (secondary outcome) was analysed using Cox proportional hazards models. When evaluating kidney failure/KRT, participants were censored on either the site-specific date that the database was updated, last date the person was seen before being lost to follow-up, or date of death. When evaluating all-cause mortality, participants were censored on the date the database was updated or date lost to follow-up. All analyses were stratified by baseline eGFR (< 45 and ≥ 45 mL/min per 1.73m^2^), which was considered a clinically significant cut-point [13]. The reference group was defined as the cohort with diabetic nephropathy as primary cause of CKD and a baseline eGFR ≥ 45 mL/min per 1.73m^2^. A sensitivity analysis evaluated time to kidney failure/KRT with death as a competing event, using Fine and Gray’s proportional sub-hazards model. Change in eGFR was analysed using multivariable linear regression, stratifying analysis by baseline eGFR. The reference group was defined as the cohort with an acquired single kidney. Unlike the other analyses, comparison was only made within instead of between eGFR strata, as the decreased accuracy of the CKD-EPI equation at lower values for serum creatinine may introduce bias if directly comparing the two strata using the same definition for eGFR change [14]. Progressive CKD was analysed using multivariable logistic regression, stratified by baseline eGFR. The reference group was defined as the cohort with diabetic nephropathy and an eGFR < 45 mL/min per 1.73m^2^. In all multivariable models, confounders were identified using directed acyclic graphs. A supplementary analysis of patients with an acquired single kidney for whom a date and indication for nephrectomy was able to be determined from chart review; indication and year were compared descriptively by whether or not the patient progressed to kidney failure. Analysis was performed using Stata 14.2 (StataCorp, College Station, TX).

## Results

This study included 4,880 general nephrology patients from Queensland, Australia (Table 1). At the time when data were extracted, 633 (12%) and 1,267 (24%) participants had developed kidney failure/commenced KRT, or died at median [interquartile range (IQR)] follow-up times of 19.2 [7.8–33.6] and 31.6 [16.0–48.9] months following recruitment. The primary indication for nephrology referral was an acquired single kidney for 235 (5%) participants; of these, 24 (10%) and 38 (17%) developed kidney failure/required KRT or died at median [IQR] follow-up times of 12.9 [2.5–31.0] and 33.6 [18.0–57.9] months, respectively, following recruitment.

**Table 1:** Baseline characteristics of study population

Variable	(*n* = 4880)
Age, years	
Median [IQR]	68.2 [56.4–76.5]
Sex (*n*, %)	
Female	2304 (47)
Male	2576 (53)
Diabetes mellitus (*n*, %)	
No	2448 (50)
Yes	2368 (49)
Missing	64 (1)
Hypertension (*n*, %)	
No	811 (17)
Yes	3516 (72)
Missing	553 (11)
Smoker status (*n*, %)	
Never	876 (18)
Former	1155 (24)
Current	452 (9)
Missing	2397 (49)
Baseline eGFR	
Median [IQR]	39 [29–54]
Range	15–94
Primary kidney disease (*n*, %)	
Diabetic nephropathy	1238 (25)
Glomerulonephritis	616 (13)
PCKD	139 (3)
Acquired single Kidney	221 (5)
Other	2666 (55)

eGFR estimated glomerular filtration rate (in mL/min per 1.72m^2^), *IQR* interquartile range, *PCKD* polycystic kidney diseases

**Fig. 1:**
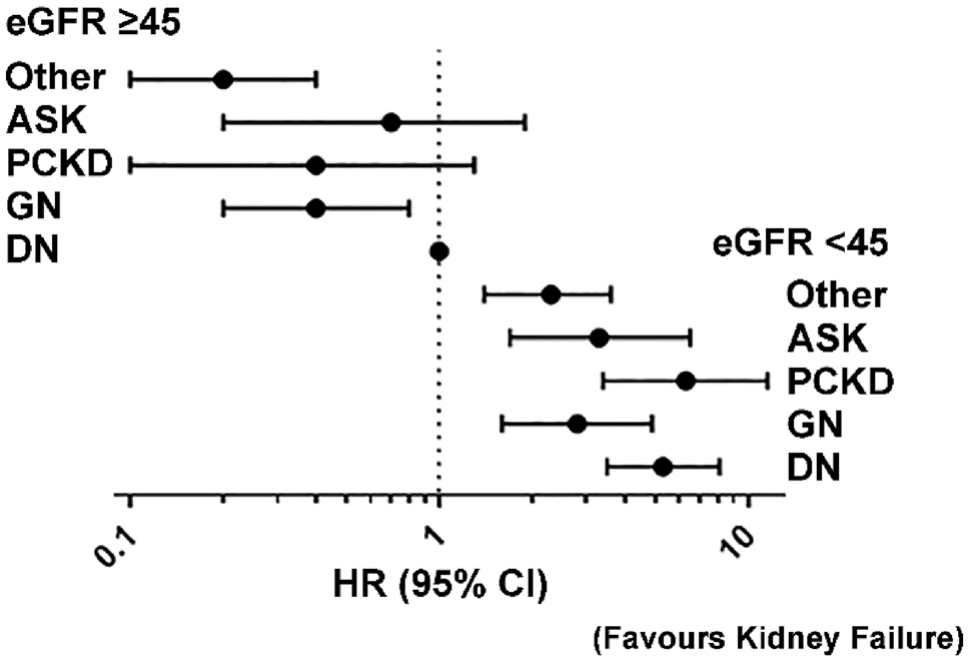
Likelihood of kidney failure compared by primary cause of CKD. Forest plot comparing the hazard ratio (HR) and 95% confidence interval (CI) for the development of kidney failure after recruitment compared by primary aetiology of chronic kidney disease (CKD). Results stratified by estimated glomerular filtration rate (eGFR; in mL/min per 1.73m^2^). *ASK* acquired single kidney; *DN* diabetic nephropathy, *GN* glomerulonephritis, *PCKD* polycystic kidney disease

Rates of kidney failure/KRT were compared by primary aetiology of CKD and dichotomised to eGFR (at recruitment) < 45 and ≥ 45 mL/min per 1.73m^2^ (Table 2; Fig. 1). Among participants with an eGFR < 45 mL/min per 1.73m^2^, those with PCKD had the highest rates (per 1,000 person-years) of kidney failure/KRT (107.8, 95% confidence interval [CI] 71.0–163.8). The diabetic nephropathy cohort had the next highest (75.5, 95%CI 65.6–87.1), followed by the glomerulonephritis cohort (52.9, 95% CI 38.8–72.1). The acquired single kidney cohort had the lowest crude rate of kidney failure (34.6, 95% CI 20.5–58.4) among aetiological groups. These rankings were preserved when evaluating point estimates for the crude hazard ratios for risk of kidney failure/KRT; however, there was no difference between the acquired single kidney and glomerulonephritis groups when adjustment was made for age, sex, and the presence of diabetes/hypertension. Among the eGFR ≥ 45 mL/min per 1.73m^2^ groups, the highest rates of kidney failure/KRT was seen in the diabetic nephropathy cohort (16.6, 95% CI 92.5–117.3), with similar rates in the glomerulonephritis, PCKD, and acquired single kidney cohorts (11.3, 95% CI 7.1–17.9; 11.7, 95% CI 3.8–36.2; 10.7, 95% CI 4.0–28.4, respectively). Crude hazard ratios for kidney failure/KRT were similar across the aetiological groups. After adjusting for age, sex, and diabetes/hypertension, the point estimate for those with an acquired single kidney was higher than all groups except diabetic nephropathy. Similar patterns were observed in the analysis considering death as a competing event (Supplementary Table 1).

**Table 2:** Kidney failure and death compared by primary kidney disease (*n* = 4,880)

	Person-years	Number of events	Rate^a^ (95% CI)	Crude HR (95% CI)	Adjusted HR (95% CI)
Kidney failure^b^					
Baseline eGFR < 45					
Diabetic nephropathy	2516	190	75.5 (65.5–87.1)	4.6 (3.0–6.9)	5.3 (3.5–8.1)
Glomerulonephritis	757	40	52.9 (38.8–72.1)	3.2 (1.9–5.2)	2.8 (1.6–4.9)
PCKD	204	22	107.8 (71.0–163.8)	6.5 (3.7–11.4)	6.3 (3.4–11.6)
Acquired single kidney	405	14	34.6 (20.5–58.4)	2.1 (1.1–4.0)	3.3 (1.7–6.5)
Other	5570	139	25.0 (21.1–29.5)	1.5 (0.9–4.0)	2.3 (1.4–3.6)
Baseline eGFR ≥ 45					
Diabetic nephropathy	1564	26	16.6 (11.3–24.4)	1.0	1.0
Glomerulonephritis	1595	18	11.3 (7.1–17.9)	0.7 (0.4–1.2)	0.4 (0.2–0.8)
PCKD	257	3	11.7 (3.8–36.2)	0.6 (0.2–1.8)	0.4 (0.1–1.3)
Acquired single kidney	375	4	10.7 (4.0–28.4)	0.6 (0.2–1.8)	0.7 (0.2–1.9)
Other	3335	12	3.6 (2.0–6.3)	0.2 (0.1–0.4)	0.2 (0.1–0.4)
Death^c^					
Baseline eGFR < 45					
Diabetic nephropathy	2630	274	104.2 (92.5–117.3)	3.0 (2.3–4.0)	2.3 (1.7–3.1)
Glomerulonephritis	782	41	52.4 (38.6–71.2)	1.4 (1.0–2.2)	2.0 (1.3–3.0)
PCKD	206	5	24.2 (10.1–58.2)	0.7 (0.3–1.8)	1.0 (0.4–2.5)
Acquired single kidney	424	30	70.7 (49.4–101.1)	2.0 (1.3–3.1)	1.5 (1.0–2.4)
Other	5.793	574	99.1 (91.3–107.6)	2.8 (2.2–3.7)	2.1 (1.6–2.8)
Baseline eGFR ≥ 45					
Diabetic nephropathy	1577	57	36.1 (27.9–46.8)	1.0	1.0
Glomerulonephritis	1605	15	9.3 (5.6–15.5)	0.2 (0.1–0.4)	0.6 (0.3–1.0)
PCKD	260	3	11.6 (3.7–35.8)	0.3 (0.1–1.1)	1.0 (0.3–3.2)
Acquired single kidney	382	8	20.9 (10.5–41.9)	0.6 (0.3–1.2)	0.9 (0.4–1.9)
Other	3377	102	30.2 (24.9–36.8)	0.8 (0.6–1.1)	1.0 (0.8–1.5)

Hazard ratios (HR) and 95% confidence intervals (CI) estimated using Cox proportional hazards models

*eGFR* estimated glomerular filtration rate (in mL/min per 1.73m^2^), *PCKD* polycystic kidney diseases

^a^Unadjusted rate per 1000 person-years

^b^Multivariable model adjusted for age, sex, diabetes mellitus, and hypertension

^c^Multivariable model adjusted for age, sex, diabetes mellitus, hypertension, and smoker status

Rates of death in participants with an acquired single kidney were 70.7 (95% CI 49.4–101.1) in the eGFR < 45 mL/min per 1.73m^2^ group, and 20.9 (95% CI 10.5–41.9) in the eGFR ≥ 45 mL/min per 1.73m^2^ group (Table 2). Differences in risk of death across the aetiological groups disappeared when assessing hazard ratios adjusted for age, sex, smoker status, and the presence of diabetes and hypertension.

There were similar relationships among the aetiological groups when evaluating CKD progression and change in eGFR as there was when evaluating kidney failure (Supplementary Table 2).

Participants in the acquired single kidney cohort who developed kidney failure/KRT tended to be male, have comorbidities, such as hypertension and diabetes mellitus, and a lower eGFR at recruitment, compared with those who did not develop kidney failure/KRT (Table 3). There was no substantial difference between indication or timing of nephrectomy and development of kidney failure (Supplementary Table 3 and 4).

**Table 3:** Baseline characteristics of patients with an acquired single kidney

Variable	Developed kidney failure		*p* value
	No (*n* = 203)	Yes (*n* = 18)	
Age, years			
Median [IQR]	67.2 [59.4–76.6]	67.1 [59.8–72.7]	0.84
Sex (*n*, %)			
Female	93 (46)	4 (22)	0.05
Male	110 (54)	14 (78)	
Diabetes mellitus (*n*, %)			
No	125 (62)	9 (50)	0.45
Yes	74 (36)	8 (44)	
Missing	4 (2)	1 (6)	
Hypertension (*n*, %)			
No	52 (26)	2 (11)	0.03
Yes	119 (59)	17 (89)	
Missing	32 (16)	–	
Smoker status (*n*, %)			
Never	37 (18)	3 (17)	0.30
Former	42 (21)	7 (39)	
Current	19 (9)	2 (11)	
Missing	105 (52)	6 (33)	
Baseline eGFR			
Median [IQR]	44 [32–59]	24 [19–41]	< 0.001
Range	15–91	15–68	

P values were estimated using either a Mann–Whitney *U*-test or Chi-square test, depending on whether continuous or categorical

*eGFR* estimated glomerular filtration rate (in mL/min per 1.72m^2^), *IQR* interquartile range; PCKD, polycystic kidney diseases

## Discussion

This study aimed to evaluate rates of kidney failure/KRT, mortality, and CKD progression in people with an acquired single kidney and managed in the clinical practices of specialist nephrologists. These were compared with other patients managed in these practices with CKD of other aetiologies.

As expected, likelihood of kidney failure/KRT was higher in the eGFR < 45 mL/min per 1.72m^2^ group compared with those with a higher baseline eGFR. Participants in the diabetic nephropathy cohort were particularly likely to reach this endpoint compared with those who had an acquired single kidney, regardless of baseline eGFR. This is not surprising, given the multiple pathophysiological mechanisms involved in diabetes mellitus, including recurrent and/or persistent hyperglycaemia, macro- and microvascular atherosclerotic changes, and cardiovascular disease, in addition to processes activated by nephron loss [15, 16].

Participants with glomerulonephritis had very similar risks of kidney failure/KRT compared with participants with an acquired single kidney. Notwithstanding, the estimates were relatively imprecise with overlapping confidence intervals between aetiologies, due in part to small sample sizes. Outcomes in the glomerular disease group are variable, due to the heterogeneity of aetiology and response to targeted therapies. The latter in particular may explain the reduced likelihood of progression in this cohort [17].

Baseline eGFR modified the effect of kidney disease aetiology on kidney failure/KRT, as demonstrated by the point estimates in the PCKD group. The PCKD cohort with a baseline eGFR < 45 ml/min per 1.72m^2^ had the highest point estimates for kidney failure/KRT compared with other CKD aetiologies; whereas the PCKD cohort in the higher eGFR group had comparatively lower point estimates compared with diabetic nephropathy, and comparable point estimates to the acquired single kidney cohort. This difference could have a biological basis, due to the multiplicity of mutations associated with PCKD, and may reflect unequal distribution of mutations between the eGFR groups thereby leading to differences in outcomes. For example, mutations at the PKD-1 locus are associated with a higher likelihood of CKD progression compared with PKD-2 [18], and it could be hypothesised that there was a higher proportion of PKD-1 mutations in the group with the lower eGFR. Genetic information was not available in this cohort, so this hypothesis could not be explored further.

Similar patterns were observed for the outcomes of CKD progression and eGFR change, as for kidney failure/KRT. The diabetic nephropathy and PCKD cohorts in the baseline eGFR < 45 mL/min per 1.73m^2^ group had higher rates of progression than other cohorts, including acquired single kidney; the remaining groups were comparable. In the eGFR ≥ 45 mL/min per 1.73m^2^ group, diabetic nephropathy was associated with higher rates of CKD progression and greater reductions in eGFR; whereas the other groups had lower and equivalent estimates.

Collectively, we found that people with diabetic nephropathy were more likely to develop kidney failure/KRT than those with CKD due to an acquired single kidney. Other non-surgical causes of CKD, such as glomerulonephritis, had rates of kidney failure/KRT and CKD progression that were similar to CKD due to an acquired single kidney. Diabetic nephropathy is the most common cause of CKD in most Western countries [19], and is likely to be disproportionately represented in analyses which combine non-surgical causes of CKD and compare these collectively with surgical causes. This would skew the effect size away from the null hypothesis, and may explain the results of previous studies which reported non-surgical causes of CKD are more likely to progress to kidney failure/KRT than surgical causes [3, 4].

Also notable was the fact that, regardless of underlying aetiology of CKD, risk of adverse outcomes increased substantially with lower eGFR (< 45 mL/min per 1.72m^2^), which is consistent with previous studies [3]. This exemplifies the point that eGFR rather than aetiology of kidney disease should be the driver for risk stratification.

In this cohort, 10% of the patients with an acquired single kidney developed kidney failure/KRT at a median 19 months after recruitment, and 17% died at a median 31 months after recruitment. These figures overestimate the expected absolute risk of kidney failure and death among patients with CKD after nephrectomy due to referral bias, and it is reasonable to expect that many patients in this cohort with an acquired single kidney had other risk factors for CKD. Nonetheless, a significant proportion of people in this group experienced adverse outcomes, which is not characteristic of a benign disease process. These results are consistent with a population-based study, also conducted in Queensland, which demonstrated that of 2,739 patients who underwent nephrectomy for kidney cancer between 2009 and 2014, 1.9% and 1.0% who underwent either radical or partial nephrectomy went on to develop incident kidney failure within three years of surgery [20].

The strengths of this study lie in its large sample size, and access to information relating to the aetiology of CKD diagnosis, which allowed for a more in-depth comparison of medical and surgical causes of CKD. Limitations include missing data regarding dates of nephrectomy or initial diagnosis of CKD, introducing risk of lead–time and length–time bias. Even with these data comparing surgical causes of CKD with other non-surgical causes, using time-to-event analysis is difficult because surgical causes have a distinct T_0_, whereas non-surgical causes rarely have a known date of diagnosis, let alone a date of onset/initiating injury. There were limited data available on comorbidities due to variable capture across the included health services, including changes in comorbidities over time, which may have impacted our analysis.

## Conclusion

This study has demonstrated that patients with CKD due to an acquired single kidney have comparable risks of kidney failure and progression to many non-surgical aetiologies of CKD. It is notable that patients with CKD due to diabetic nephropathy who are known to specialist nephrology practices generally had higher likelihood of kidney failure, CKD progression and death, compared with patients who had CKD due to an acquired single kidney. It is likely that the results from previous studies that demonstrate medical causes of CKD as being more aggressive than surgical causes are influenced by the high proportions of patients with diabetic nephropathy in the medical CKD cohorts, which may downplay the risk of surgical CKD. While CKD after nephrectomy is not the most aggressive cause of kidney disease, it is by no means benign, and among patients known to specialist nephrology practices in Queensland, it is associated with a tangible risk of kidney failure and death, that is comparable to other major causes of CKD.

## Supplementary Information

Below is the link to the electronic supplementary material.

Supplementary file1 (DOCX 21 KB)

## Data Availability

Data can be obtained on application to the CKD.QLD registry, with appropriate permissions and ethics approvals obtained.

## Code availability

Data were analysed using Stata 14.2 (StataCorp, College Station, TX) using standard code.
