# Supplementary material for: Kidney failure, CKD progression and mortality after nephrectomy
Source: Int Urol Nephrol. 2022 Jan 27;54(9):2239–45. doi: 10.1007/s11255-022-03114-7 (PMC9371989; doi:10.1007/s11255-022-03114-7)
Supplement: Supplementary file 1 — Supplementary file1 (DOCX 21 KB) [file 11255_2022_3114_MOESM1_ESM.docx]

| **Supplementary Table 1** – Incidence of kidney failure considering death as a competing event (n = 4,880) | | | | | | |
| --- | --- | --- | --- | --- | --- | --- |
|  | | Person-Years | Number of Events | Rate ^a^ (95% CI) | Crude SHR (95% CI) | Adjusted ^b^ SHR (95% CI) |
| *Baseline eGFR <45* | |  |  |  |  |  |
|  | Diabetic nephropathy | 2,516 | 190 | 75.5 (65.5-87.1) | 4.0 (2.7-6.1) | 5.0 (3.3-7.4) |
|  | Glomerulonephritis | 757 | 40 | 52.9 (38.8-72.1) | 3.1 (1.9-5.0) | 2.7 (1.6-4.6) |
|  | PCKD | 204 | 22 | 107.8 (71.0-163.8) | 6.7 (3.9-11.5) | 6.1 (3.4-11.1) |
|  | Acquired Single Kidney | 405 | 14 | 34.6 (20.5-58.4) | 2.0 (1.0-3.8) | 3.3 (1.7-6.4) |
|  | Other | 5,570 | 139 | 25.0 (21.1-29.5) | 1.3 (0.9-2.0) | 2.1 (1.3-3.2) |
| *Baseline eGFR ≥45* | |  |  |  |  |  |
|  | Diabetic nephropathy | 1,564 | 26 | 16.6 (11.3-24.4) | 1.0 | 1.0 |
|  | Glomerulonephritis | 1,595 | 18 | 11.3 (7.1-17.9) | 0.7 (0.4-1.3) | 0.4 (0.2-0.8) |
|  | PCKD | 257 | 3 | 11.7 (3.8-36.2) | 0.7 (0.2-2.3) | 0.3 (0.1-1.2) |
|  | Acquired Single Kidney | 375 | 4 | 10.7 (4.0-28.4) | 0.7 (0.2-1.9) | 0.6 (0.2-2.0) |
|  | Other | 3,335 | 12 | 3.6 (2.0-6.3) | 0.2 (0.1-0.4) | 0.2 (0.1-0.4) |
| Subhazard ratios (SHR) and 95% confidence intervals (CI) estimated using Fine and Gray’s proportional subhazards model.  ^a^ Unadjusted rate per 1,000 person-years.  ^b^ Multivariable model adjusted for age, sex, diabetes mellitus, and hypertension.  Abbreviations: eGFR, estimated glomerular filtration rate (in mL/min per 1.73m^2^); PCKD, polycystic kidney diseases. | | | | | | |

| **Supplementary Table 2** – Decline in eGFR and CKD progression compared by primary kidney disease | | | | | | |
| --- | --- | --- | --- | --- | --- | --- |
|  |  | *n* (%) | Baseline | Final | Adjusted β | Adjusted OR |
|  |  |  | eGFR ^a^ | eGFR ^b^ | (95% CI) ^c^ | (95% CI) ^d^ |
| *Baseline eGFR <45* | |  |  |  |  |  |
|  | Diabetic nephropathy | 597 | 29 [22-36] | 26 [18-34] | -2.1 (-4.7 to 0.4) | 1.00 |
|  | Glomerulonephritis | 166 | 33 [23-38] | 32 [23-43] | 3.8 (0.9 to 6.7) | 0.5 (0.3-0.7) |
|  | PCKD | 54 | 30 [23-35] | 27 [18-35] | -2.8 (-6.6 to 1.0) | 1.1 (0.6-1.9) |
|  | Acquired Single Kidney | 86 | 32 [26-38] | 32 [23-38] | 0.00 | 0.5 (0.3-0.9) |
|  | Other | 1,293 | 31 [25-37] | 30 [22-39] | 0.4 (-2.0 to 2.8) | 0.5 (0.4-0.6) |
| *Baseline eGFR ≥45* | |  |  |  |  |  |
|  | Diabetic nephropathy | 235 | 54 [49-68] | 52 [40-65] | -4.3 (-8.1 to -0.4) | 1.1 (0.8-1.5) |
|  | Glomerulonephritis | 268 | 78 [59-91] | 79 [55-90] | -1.8 (-5.4 to 1.8) | 0.6 (0.4-0.8) |
|  | PCKD | 53 | 73 [57-90] | 72 [54-90] | -2.0 (-6.9 to 2.8) | 0.3 (0.2-0.6) |
|  | Acquired Single Kidney | 72 | 59 [50-68] | 58 [49-71] | 0.00 | 0.4 (0.3-0.7) |
|  | Other | 560 | 55 [49-67] | 56 [47-70] | 0.3 (-3.0 to 3.7) | 0.6 (0.5-0.8) |
| ^a^ Baseline eGFR recorded at the time of recruitment, presented as median [interquartile range].  ^b^ Final eGFR recorded at either 12 or 24 months following recruitment, presented as median [interquartile range].  ^c^ Change in eGFR between baseline and final measurement compared by primary kidney disease. A positive coefficient indicates a smaller eGFR decline compared with patients with an acquired single kidney, and a negative coefficient indicates a larger eGFR decline (n = 3,384). Beta coefficient (β) and 95% confidence interval (CI) estimated using a multivariable linear regression model, adjusted for age, sex, diabetes mellitus, and hypertension.  ^d^ CKD progression, defined as an annual eGFR decline of ≥5 mL/min per 1.73m^2^ in at least one year without subsequent recovery, or the development of kidney failure or requirement for kidney replacement therapy within the two year period following recruitment (n = 3,463). Odds ratio (OR) and 95% CI estimated using a multivariable logistic regression model, adjusted for age, sex, diabetes mellitus, hypertension, and smoker status.  Abbreviations: CKD, chronic kidney disease; eGFR, estimated glomerular filtration rate (in mL/min per 1.73m^2^); PCKD, polycystic kidney diseases. | | | | | | |

| **Supplementary Table 3** – Kidney failure in patients with an acquired single kidney | | | | |
| --- | --- | --- | --- | --- |
|  | | Kidney Failure | | p-value |
|  |  | No | Yes |  |
| Age | |  |  |  |
|  | <65 | 60 (39) | 9 (53) | 0.26 |
|  | ≥65 | 95 (61) | 8 (47) |  |
| Sex | |  |  |  |
|  | Female | 67 (43) | 6 (35) | 0.53 |
|  | Male | 88 (57) | 11 (65) |  |
| Indication | |  |  |  |
|  | Tumour | 112 (72) | 14 (82) | 0.67 |
|  | Living Donor | 14 (93) | 1 (6) |  |
|  | Other | 29 (94) | 2 (12) |  |
| Year of Surgery | |  |  |  |
|  | < 1999 | 35 (23) | 5 (29) | 0.76 |
|  | 2000-2009 | 57 (37) | 5 (29) |  |
|  | 2010-2020 | 63 (42) | 7 (41) |  |
| Proportion of patients who did and did not develop kidney failure in a subset of 172 patients in the acquired single kidney group for whom the indication for and date of nephrectomy were able to be determined from chart review. P-values estimated using a Chi-square test. | | | | |

| **Supplementary Table 4** – Kidney failure in patients with an acquired single kidney by aetiology | | | |
| --- | --- | --- | --- |
|  | | Kidney Failure | |
|  |  | No | Yes |
| Indication | |  |  |
|  | RCC | 86 (55) | 12 (71) |
|  | TCC | 18 (12) | 2 (2) |
|  | Benign tumour | 4 (3) | - |
|  | Other malignancy | 4 (3) | - |
|  | Living kidney donor | 14 (9) | 1 (6) |
|  | Obstruction/Stones | 11 (7) | 1 (6) |
|  | Trauma | 5 (3) | 1 (6) |
|  | Infection | 4 (3) | - |
|  | Other | 9 (6) | - |
| Proportion of patients who did and did not develop kidney failure in a subset of 172 patients in the acquired single kidney group for whom the indication for and date of nephrectomy were able to be determined from chart review. RCC, renal cell carcinoma; TCC, transitional cell carcinoma. | | | |
